# An In Vitro Exploration of Interaction Mechanisms of Intracoronal Bleaching on the Compressive Strength of Conventional and Calcium Silicate–Based Self-Adhesive Resins and Their Bonding to Composite Resin Restorative Material

**DOI:** 10.1155/2024/6645237

**Published:** 2024-10-22

**Authors:** Fereshteh Shafiei, Paria Dehghanian, Shadi Tivay, Yasamin Ghahramani

**Affiliations:** ^1^Oral and Dental Disease Research Center, Department of Operative Dentistry, School of Dentistry, Shiraz University of Medical Sciences, Shiraz, Iran; ^2^Oral and Dental Disease Research Center, Department of Operative Dentistry, Shiraz University of Medical Sciences, Shiraz, Iran; ^3^Operative Dentistry, Department of Operative Dentistry, School of Dentistry, Shiraz University of Medical Sciences, Shiraz, Iran; ^4^Oral and Dental Disease Research Center, Department of Endodontics, School of Dentistry, Shiraz University of Medical Sciences, Shiraz, Iran

## Abstract

**Objectives:** The cervical resorption following intracoronal bleaching necessitates the application of impermeable cervical barriers. This study aimed to evaluate the effect of two bleaching agents on the compressive strength (CS) and shear bond strength (SBS) of two self-adhesive resins, TheraCem and Vertise Flow, to composite resin restorative material.

**Materials and Methods:** Two hundred sixteen specimens from TheraCem and Vertise Flow were prepared in special molds and treated in three groups: nonbleached (control); sodium perborate–hydrogen peroxide (SP–HP) (sodium perborate +3% hydrogen peroxide); and HP gel (35% hydrogen peroxide gel). The CS of 72 specimens in the three groups was tested using a universal testing machine. For SBS test, 144 specimens from TheraCem and Vertise Flow in the three groups were bonded to Z250 composite using Single Bond Universal adhesive in self-etch and etch-and-rinse modes. SBS was measured using universal testing machine. Data were analyzed using two-way ANOVA and Tukey tests for CS and three-way ANOVA and Tukey tests for SBS.

**Results:** Vertise Flow showed higher CS than TheraCem (*p* < 0.001), while none of the bleaching agents deteriorated its CS. HP gel significantly lowered the CS of TheraCem (*p* = 0.001). Bleaching agents increased the SBS of Vertise Flow while the SBS of TheraCem was not significantly affected. For both resin barriers, SBS was higher in the etch-and-rinse mode (*p* < 0.05), except in nonbleached Vertise Flow (*p* = 0.091).

**Conclusions:** HP gel deleteriously affected the CS of only TheraCem during nonvital bleaching. The etch-and-rinse mode was preferred in terms of SBS for resin barriers.

## 1. Introduction

Bleaching is a conservative, affordable, and effective method for the esthetic treatment of discolored endodontically treated teeth [1]. This treatment, also known as nonvital or walking bleaching, involves tooth whitening through applying peroxide-based agents in the pulp chamber to oxidize discolored molecules and break the bonds that hold them together [2]. The most commonly used bleaching agents include hydrogen peroxide (HP) in various concentrations. Others, like sodium perborate (SP), release HP and can be used either in its pure form or mixed with 30% HP or distilled water. Additionally, carbamide peroxide (CP), also known as urea peroxide, can be utilized either on its own or in combination with other substances [3, 4].

One of the most serious complications that can arise after this treatment is external root resorption, which may occur due to various factors, including material properties, technique-related errors, or dental trauma.

Cervical resorption could be a particularly harmful side effect of nonvital bleaching, due to the formation of an acidic condition at the cervical area that leads to increased activity of osteoclasts [5]. Therefore, using an impermeable cervical barrier to prevent the leakage of the bleaching agent and seal the root canal filling before the bleaching procedure is highly recommended [1, 5]. However, a completely impermeable bonding interface has not still achieved. Applying a 2–3 mm thickness of cervical barrier not only protects the root canal filling but also serves as a base for the final restoration after the bleaching procedure. Consequently, the ideal barrier's sealing ability and mechanical properties should not be negatively affected by the bleaching process [6]. To fulfill these criteria, various materials have been suggested. Glass ionomer (GI), especially light-cured resin-modified GI, has been widely used as the dressing material for many years [5, 7].

Calcium silicate cements such as mineral trioxide aggregate (MTA) and Biodentine are raised as intraorifice barriers that not only produce sufficient cervical sealing but also have alkaline properties to minimize cervical resorption [8, 9]. In Canoglu et al.'s [10] examination, MTA showed the best sealing ability followed by resin-based composite while the GI barrier showed the greatest amount of leakage. Nevertheless, MTA and Biodentine are not clinically easy to handle. Clinicians would hardly turn a blind eye to their prolonged setting time and tooth discoloration. In fact, intracoronal bleaching is performed to lighten tooth darkness and if the cervical barrier produces new discolorations, it contradicts the bleaching goals. According to Keskin et al.'s recent study, the combination of SP and HP would reduce the compressive strength (CS) of Biodentine and three kinds of MTA. Surface cracks and a decrease in bond strength of composite to Biodentine following bleaching have been reported as well. The resulting surface cracks may lead to microleakage and the inability of MTA and Biodentine to provide an effective barrier. Bleaching agents were reported to adversely affect surface microhardness, surface roughness, and elemental distribution of MTA [11]. Therefore, using a resin layer to protect calcium silicate cements prior to the bleaching procedure is suggested [12–14]. Accordingly, the use of resin composite as a cervical base, while creating sufficient sealing, allows the clinician to perform the bleaching procedure in the same session. Also, it does not cause any changes in the tooth color [10, 15]. Self-adhesive resin materials such as Vertise Flow simplify the bonding procedure and reduce technique sensitivity [16, 17], rendering them desirable base materials. However, Vertise Flow (being a flowable resin composite) has no defensive effect against resorption.

Another self-adhesive resin, TheraCem (Bisco, United States), is a calcium silicate–based resin cement known for its clinical ease of use due to self-adhesiveness. Its stable bond in acidic environments, alkalinity after setting (pH = 9), and calcium release make it a favorable bioactive alternative for MTA and Biodentine [18, 19]. Furthermore, these properties could offer a protective effect against cervical resorption.

After the bleaching was completed, using a conventional composite along with a universal adhesive could ensure a stable and durable restoration for the bleached teeth. While self-adhesive flowable composite resins are formulated with hydrophilic monomers, conventional composites are devoid of such elements. This results in greater stability of the mechanical properties of conventional composites after aging [20].

Universal adhesives, featuring a newer adhesive system, could bond effectively to various substrates, including enamel, dentin, and composite resin. They are used in etch and rinse mode, self-etch mode, and selective enamel etch mode [21]. An earlier study demonstrated the negative impact of bleaching agents on the bond strength between calcium silicate–based cement (Biodentine) and composite resin when using an etch-and-rinse adhesive [13]. Bleaching agents may produce alterations in resin barriers, affecting composite resin bond strength.

Achieving proper bonding of resin composites to the cervical base after the bleaching process is essential for successful treatment. In addition, bleaching should not compromise the mechanical strength of the protective base. To the best of our knowledge, no previous studies have evaluated the effect of two bleaching agents on the CS of TheraCem and Vertise Flow, nor their bonding ability to conventional composites in the internal bleaching procedure. The purpose of this study is to evaluate the null hypotheses that nonvital bleaching agents would not alter the CS of the self-adhesive resin cement (TheraCem) and the flowable self-adhesive composite resin (Vertise Flow); and their bond strength to a universal adhesive/resin-composite would not be affected in the two bonding approaches.

## 2. Materials and Methods

**Figure 1:**
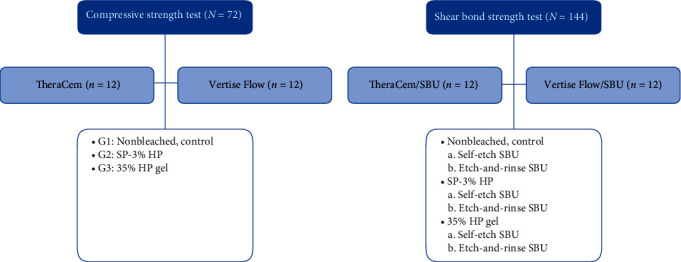
Flowchart of the study groups. HP, hydrogen peroxide; SBU, Single Bond Universal; SP, sodium perborate.

In this experimental study, a self-adhesive resin cement (TheraCem, Bisco, United States) and a self-adhesive flowable composite resin (Vertise Flow; Kerr Dental, Italy) were evaluated in terms of CS and shear bond strength (SBS) of Single Bond Universal (SBU) (SBU, 3M ESPE, St. Paul, MN, United States) in different bleaching agents and acid-etching modes. As the study groups are shown in Figure 1, the type of resin base and bleaching agent were independent variables, while the CS was the dependent variable. Additionally, the type of resin base, bleaching agent, and bonding technique were independent variables, and the SBS was the dependent variable.  Table 1 shows the materials used in this study.

### 2.1. CS Test

A total of 72 disc-shaped specimens (6 mm in height and 4 mm in diameter) were prepared from each of TheraCem and Vertise Flow resins using split Teflon molds. The specimen dimensions were determined with a digital caliper (Mitutoyo Digimatic; Mitutoyo, Kawasaki, Japan). The resins were placed incrementally in 2 mm layers in a metal container and the first layer was cured using a light-curing unit (VIP Junior, Bisco) at a light intensity of 600 mW/cm^2^ for 20 s. The irradiance of the device was checked using a radiometer during the experiment. After insertion of the second layer, a Mylar strip and glass slab were placed on the top of the layer and light cured. Then, any resin flashes were removed using 400-grit sandpaper. The specimens of both resins were randomly divided into three groups (*n* = 12):

• G1: nonbleached; control.
• G2: SP-HP; mixture of SP powder (2 g) and 3% HP solution (1 mL) in a creamy consistency.
• G3: HP gel; 35% HP (H_2_O_2_) gel (Opalescence Endo, Ultradent, South Jordan, UT, United States).

The bleaching materials were applied on the surface of the resins with a thickness of 2 mm. Afterward, all the samples were individually transferred into custom-made silicone containers to trap the bleaching materials and incubated at 37°C and relative 95% humidity for 1 weak [12, 13, 22]. After that, the exposed surfaces of the samples were rinsed with 5 mL of distilled water for 1 min and gently air dried with an air syringe, according to Sismanoglu et al.'s [22] study. Eventually, the CS was tested in all of the groups using a universal testing machine (Zwick, Ulm, Germany) at a crosshead speed of 1 mm/min. CS in MPa was measured by dividing the maximum load of fracture in newton by the diameter of the specimen (in millimeters) using the following equation:

  $$\begin{array}{c} \text{CS }=\frac{4P}{\pi d^{2}}. \end{array}$$

### 2.2. SBS Test

One hundred forty-four cylindrical specimens of each self-adhesive resin (TheraCem and Vertise Flow) were fabricated by filling them into plastic molds, measuring 4 mm in internal diameter and 2 mm in height. To ensure consistency, the resins were light cured for 20 s using the light-curing unit. Subsequently, all specimens were polished to achieve a standardized flat surface, employing 400-grit silicon carbide abrasive paper for a duration of 60 s. After washing and drying, an adhesive tape with a 4-mm diameter in it was used to define the bonding area. They were then divided into three groups including nonbleached, HP gel, and SP–HP that were applied for 7 days, as mentioned earlier. The bonding procedures were performed in all groups using SBU adhesive in two strategies. In etch-and-rinse mode, the surface was acid etched using 35% phosphoric acid for 15 s, washed for 20 s, and air dried to remove excess water. The adhesive was applied with a microbrush in two layers for 10–15 s and gently air dried. The adhesive was light cured. In self-etch mode, the adhesive was applied on the surface without acid etching as mentioned earlier. Subsequently, a cylindrical plastic mold, measuring 2 mm in internal diameter and 3 mm in height, was installed on the testing samples to characterize the bonded specimens. Plastic molds were then filled with Z250 composite (Filtek Z250, 3M ESPE, Seefeld, Germany) in two layers and light cured for 40 s. After storage in distilled water for 24 h at 37°C, the mold was carefully removed using a sharp scalpel, and the SBS was tested using the same machine at a crosshead speed of 1 mm/min. The values were calculated in MPa by dividing the peak load of failure in Newton by the specimen surface area using the following equation:

  $$\begin{array}{c} \text{SBS}=\frac{F}{\pi r^{2}}. \end{array}$$

Note that “*r”* represents one-half of the diameter of the specimen in millimeters.

All the specimen preparations were performed by a specialist in operative dentistry. Failure modes were examined under a stereomicroscope at ×20 magnification and classified into three types: adhesive failure at the interface between resin barrier and composite resin, cohesive failure within resin barrier or composite resin, and mixed failure, combination of adhesive and cohesive failure of the resin materials.

### 2.3. Scanning Electron Microscopy (SEM)

Three additional specimens from each group for both of the self-adhesive materials were prepared as described in the section of CS test. The specimens were observed using SEM (TESCAN UK VEGA III, England, 20 kV accelerating voltage, ×500 magnification) for microstructure alterations affected by the bleaching agents used.

### 2.4. Statistical Analysis

After verifying the homogeneity of the data distribution using a normality test (Kolmogorov–Smirnov test), the data were analyzed using two-way ANOVA and Tukey tests for CS and three-way ANOVA and Tukey tests for SBS. The significance level was *p* < 0.05. All statistical analyses were performed using the SPSS software, Version 16.0 (SPSS Inc, Chicago, IL, United States).

## 3. Results

The means and standard deviations of the CS and SBS values in all groups are presented in Tables 2 and 3, respectively.

The results of the two-way ANOVA test for CS are shown in Table 4. They revealed a statistically significant difference between the two types of resin bases (*p* < 0.001) and various bleaching agents (*p* = 0.001). In addition, the interactions between these two factors were statistically significant (*p* = 0.043). CS results revealed that Vertise Flow had a significantly higher CS than TheraCem, regardless of the bleaching method applied. Unlike TheraCem, none of the bleaching methods statistically deteriorated the CS of Vertise Flow (*p* = 0.202). HP gel noticeably lowered the CS of TheraCem unlike other bleaching conditions (*p* = 0.002). However, nonbleached and SP–HP specimens did not produce different results for TheraCem (*p* = 0.978).

The results of the three-way ANOVA test for SBS are presented in Table 5. They revealed that the effect of a resin base, bleaching agent, and bonding technique was significant (*p* < 0.001, *p* = 0.038, and *p* < 0.001, respectively). The interactions between resin and bleaching (*p* = 0.041), between bleaching and bonding technique (*p* = 0.012), and between resin and bonding technique (*p* < 0.001) were statistically significant. In addition, the interaction between resin, bonding agent, and bonding technique was significant (*p* = 0.032).

The results of SBS revealed that neither of the two bleaching agents affected the SBS of TheraCem in both etching modes. However, the two bleaching agents significantly increased the SBS of Vertise Flow in the etch-and-rinse mode (*p* < 0.02). In the groups in which SBU was applied in self-etch mode, Vertise Flow exhibited a higher SBS than TheraCem (*p* < 0.05). For both resins, the etch-and-rinse group for two bleaching agents had a higher SBS than that of the self-etch mode (*p* < 0.05). In two nonbleached groups of TheraCem, the etch-and-rinse mode group revealed a higher SBS than that of self-etch mode group (*p* < 0.001). This comparison for Vertise Flow was not significant (*p* = 0.091).

The evaluation of the bonded samples revealed that mixed failure was the predominant type of failure for all study groups (Table 6).

**Figure 2:**
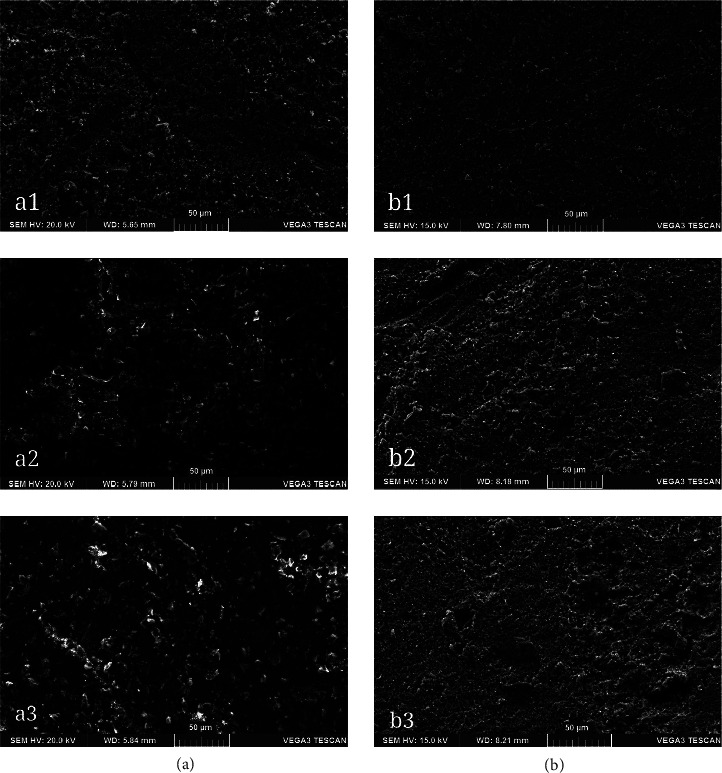
SEM images of two resin materials surface. (a) TheraCem ((a1) nonbleached; (a2) sodium perborate +3% hydrogen peroxide; and (a3) 35% hydrogen peroxide gel), (b) Vertise Flow ((b1) nonbleached; (b2) sodium perborate +3% hydrogen peroxide; and (b3) 35% hydrogen peroxide gel).

Figure 2 shows the SEM photomicrographs of the specimens' alterations caused by the bleaching agents. In the images of TheraCem specimens, slight surface deterioration was observed for SP-HP samples, while the surface alterations were more evident for HP gel specimens. The observed alterations included matrix dissolution and, to a lesser degree, exposed calcium silicate particles. The SEM observations indicated no distinctive deteriorations on the surface of Vertise Flow affected by the two bleaching agents.

## 4. Discussion

Successful nonvital bleaching is achieved by reversing the discoloration of endodontically treated teeth using HP along with an effective leak-free cervical barrier. Following the bleaching treatment, an adhesive restoration with an adequate bond strength (both to the tooth structure and the cervical barrier) can provide a desirable final treatment [13]. In the current study, a conventional composite along with a universal adhesive was used as a final restoration. However, the mechanical properties of the remaining base should not be adversely affected by the bleaching agents. In this regard, the CS and the adhesive bond strength of TheraCem and Vertise Flow were assessed after being affected by two different bleaching agents: 35%HP gel as an aggressive agent for severe discoloration, and SP + 3%HP as a mild and safe agent [23]. Overall, HP gel showed a considerable adverse influence on the CS of TheraCem, whereas SP + HP did not exert any effect on the CS of TheraCem. Neither of the agents altered the CS of Vertise Flow. Therefore, the first part of the null hypothesis was accepted for TheraCem and rejected for Vertise Flow.

The low pH and oxidizing effect of the bleaching agents have been reported to correlate with their negative effect on resin cements [24]. The effect of bleaching on the CS of resins was bleach and material dependent; the acidic pH of 5 and strong oxidizing effect of the 35%HP gel compared to the neutral pH of 7 and mild oxidizing ability of SP + 3%HP could explain the deleterious effects of HP gel on TheraCem [25, 26].

Previous reports indicated that with increasing release of H_2_O_2_ using 35%HP and CP, more crack formation occurred on the Biodentine surface as a cervical barrier [14]. Serin Kalay [11] evaluated the effect of three bleaching agents on surface properties of MTA and reported that SP caused fewer changes than CP and HP. Also, nonvital bleaching with SP + 30%HP decreased the CS of four calcium silicate–based materials. Although it is expected that resin materials are more resistant to bleaching [6], some studies reported structural alteration, increased surface roughness, and decreased mechanical properties of various resin-based materials affected by bleaching, and more so for polyacid-modified composites [27, 28].

In the cited studies, the bleaching agents were used to simulate vital bleaching (in-office and at-home) protocols in terms of concentration and application time [6, 28]. Both factors, along with monomer composition, were correlated to the different effects of bleaching agents on resin materials [27, 29]. Up to now, no study has been conducted regarding the influence of nonvital bleaching on self-adhesive resins.

In the current study, the 35% HP gel adversely affected TheraCem. However, no effect was indicated on Vertise Flow. This difference can be justified by the higher concentration of H_2_O_2_ and the higher vulnerability of TheraCem's resin matrix content to the oxidizing effect of H_2_O_2_. H_2_O_2_, with its high diffusion ability, induces oxidation cleavage in poorly polymerized chains, resulting in reduced resin microhardness [30]. Furthermore, the greater degradation susceptibility of TheraCem in the HP gel-induced acidic environment can be attributed to its composition. Since TheraCem is a self-adhesive cement that contains 10-methacryloyloxydecyl dihydrogen phosphate (MDP), the instability of the MDP molecule in acidic environments can cause hydrolytic degradation [31]. Moreover, similar to other calcium silicate–based cements, the calcium silicate component in TheraCem is prone to deterioration in acidic conditions [32]. The amount of inorganic fillers is another factor responsible for the mechanical properties of resin materials: The amount of silanized fillers in Vertise Flow is 70 wt% [33], while the amount in TheraCem is lower, at 62 wt% [19], which justifies the inferior CS of TheraCem in comparison to that of Vertise Flow.

Despite the slight surface deteriorative effects of bleaching on TheraCem in our SEM observations, its adhesive bond strength remained unaffected. In the case of Vertise Flow, the bleaching agents were found to increase the bond strength of SBU in the etch-and-rinse mode. Therefore, the second part of the tested null hypothesis was also partially rejected. However, Kucukkaya Eren et al. [13] reported that high concentrations of H_2_O_2_ produced cracks and holes in calcium silicate–based cement (Biodentine). Similarly, bleaching agents adversely affected the surface properties and elemental distribution of MTA [11]. These alterations could interfere with the mechanical properties of the cement, also negatively impacting the resin adhesive-Biodentine/MTA bond strength [11, 14]. However, the results of our SEM images did not reveal extensively damaged or cracked surfaces. This difference can be attributed to the varying compositions of the materials used in our study compared to those in the base materials of the previous study. In agreement with these findings, recently Sismanoglu et al. [22] reported superficial dissolution and surface morphology alterations of MTA cements induced by HP 35% and CP 37%. A significant reduction in their bond strength to composite resin was also demonstrated [22]. In their study, a universal adhesive was used in self-etch mode to avoid allowing the acid-etching step to mask the effect of intracoronal bleaching agents on MTA barriers [22]. Accordingly, in the current study, a universal adhesive with two etching modes was used to bond composite resin. The findings of our SBS testing indicated that the composite resin bond strength to both resin barriers in the etch-and-rinse mode was higher than in the self-etch mode when using the applied universal adhesive. One possible explanation for this finding is that the acid-etching step removed the remaining oxygen on the surface layer of the resin barrier. This could lead to closer contact between resin materials, establishing bonding between the Vertise Flow and resin composite. However, in the self-etch group, residual oxygen might linger within the surface porosities, potentially disrupting the bonding at the barrier-resin composite interface. Overall, it was reported that acid etching increases surface porosities and enhances the wettability of the MTA surface, thereby enhancing adhesive resin penetration [34].

In clinical situations where clinicians decide to use 35% HP gel for nonvital bleaching, it might be recommended that Vertise Flow act as an effective barrier due to its lower degradation compared to TheraCem. Furthermore, when it comes to the permanent restoration of bleached teeth, our research indicates the etch-and-rinse mode may be the preferred approach for universal bonding systems. Since mild universal adhesive was used in this study, it is important to note that other adhesives with different pH and composition may yield different results.

In this study, the effect of bleaching agents on the CS of two resins was assessed. It is possible that bleaching agents may also affect other mechanical properties and surface microhardness. This study was conducted in an in vitro setting that does not closely mimic the intraoral condition. The absence of thermomechanical aging procedures, which could affect the results of bond strength, was considered a limitation of the current study. These conditions should be addressed in future studies. The evaluation of other universal adhesives on resin bond strength and the impact of bleaching agents on various mechanical properties and the composition of the resin bases should be assessed in long-term studies.

## 5. Conclusion

The findings of this study revealed that the high concentration of H_2_O_2_ in the 35%HP gel diminished the CS of TheraCem but not the CS of Vertise Flow as cervical barriers. Following intracoronal bleaching, the etch-and-rinse mode of the SBU adhesive was preferred to the self-etch mode due to providing higher resin bond strength.

## Figures and Tables

**Table 1:** Materials used in this study.

Materials	Manufacturer	Type	Composition
TheraCem	Bisco, United States	Alkaline dual-cure resin cement	*Matrix:* Bisphenol-A Glycidylmethacrylate, 2-hydroxyethyl methacrylate, 10-methacryloyloxydecyl dihydrogen phosphate (10-MDP), initiators *Fillers:* 62% by weight. Glass filler, silica amorphous, ytterbium fluoride, calcium base filler, amorphous silica

Vertise Flow	Kerr dental, Italy	Light-cure self-adhering flowable composite	*Matrix*: Glycerol phosphate dimethacrylate, urethane dimethacrylate, Bisphenol-A Glycidyl methacrylate, and other methacrylate comonomers, photoinitiators *Fillers*: 70% by weight. Ytterbium fluoride, barium aluminosilicate glass, prepolymerized fillers, and colloidal silica

Single Bond Universal	3M ESPE, St. Paul, MN, United States	Light-cure universal adhesive	Methacryloyloxydecyl dihydrogen phosphate monomer, Vitrebond copolymer, dimethacrylate resins, hydroxyethyl methacrylate, filler, ethanol, water, initiators, silane

Opalescence Endo	Ultradent, South Jordan, UT, United States	Nonvital bleaching gel	Hydrogen peroxide 35%, potassium nitrate, fluoride

**Table 2:** Compressive strength values (MPa, means ± standard deviations) of TheraCem and Vertise Flow affected by intracoronal bleaching agents (*n* = 12).

Groups	TheraCem mean (MPa ± SD)*⁣*^**∗**^	Vertise flow mean (MPa ± SD)*⁣*^**∗**^
Nonbleached	151.50 (±14.60)^Aa^	263.66 (±9.62)^Ab^
SP-3%HP	150.16 (±18.27)^Aa^	254.58 (±15.71)^Ab^
35% HP gel	128.00 (±15.20)^Ba^	254.50 (±15.99)^Ab^

*Note:* Different superscript capital letters in each column and lowercase letters in each row indicate statistically significant difference.

Abbreviations: HP, hydrogen peroxide; SP, sodium perborate.

*⁣* ^**∗**^ *p* < 0.05.

**Table 3:** Shear bond strength values (MPa, means ± standard deviations) of TheraCem and Vertise Flow to Single Bond Universal adhesive affected by intracoronal bleaching agents (*n* = 12).

Groups	TheraCem mean (MPa ± SD)*⁣*^**∗**^	Vertise flow mean (MPa ± SD)*⁣*^**∗**^
Nonbleached/self-etch	9.03 (±1.07)^Aa^	12.60 (±1.27)^Ab^
Nonbleached/etch-and-rinse	14.02 (±0.88)^Ba^	13.33 (±1.26)^Aa^
SP-3%HP/self-etch	9.65 (±1.40)^Aa^	12.80 (±0.93)^Ab^
SP-3%HP/etch-and-rinse	14.29 (±2.43)^Ba^	14.20 (±1.11)^Ba^
35%HP gel/self-etch	9.77 (±1.80)^Aa^	12.83 (±1.37)^Ab^
35%HP gel/etch-and-rinse	14.49 (±1.87)^Ba^	15.11 (±3.84)^Ba^

*Note:* Different superscript capital letters in each column and lowercase letters in each row indicate statistically significant difference.

Abbreviations: HP, hydrogen peroxide; SP, sodium perborate.

*⁣* ^**∗**^ *p* < 0.05.

**Table 4:** The results of the two-way ANOVA test for compressive strength.

Source	Type III sum of squares	*df*	Mean square	*F*	*p*
Corrected mode	240,260.069	5	48,052.014	209.900	0.000
Intercept	2,891,611.681	1	2,891,611.681	12,631.095	0.000
Resin	235,412.347	1	235,412.347	1028.325	0.000
Bleaching	3341.361	2	1670.681	7.298	0.001
Resin × bleaching	1506.361	2	753.181	3.290	0.043
Error	15,109.250	66	228.928	—	—
Total	3,146,981.000	72	—	—	—
Corrected total	255,369.319	71	—	—	—

*Note:p* < 0.05 denotes statistically significant difference.

**Table 5:** The results of the three-way ANOVA test for shear bond strength.

Source	Type III sum of squares	*df*	Mean square	*F*	*p*
Corrected mode	505.053	11	45.914	25.223	0.000
Intercept	23,431.956	1	23,431.956	12,872.355	0.000
Resin	86.738	1	86.738	47.650	0.000
Bleaching	8.877	2	4.439	2.438	0.038
Bonding	319.277	1	319.277	175.395	0.000
Resin × bleaching	7.063	2	3.531	1.940	0.041
Bleaching × bonding	7.662	2	3.831	2.104	0.012
Resin × bonding	71.910	1	71.910	39.504	0.000
Error	240.284	132	1.820	—	—
Total	241,177.292	144	—	—	—
Corrected total	745.337	143	—	—	—

*Note:p* < 0.05 denotes statistically significant difference.

**Table 6:** Failure modes of the tested experimental groups.

Groups	Failure mode (adhesive/cohesive/mixed)	
	TheraCem	Vertise flow
Nonbleached/self-etch Nonbleached/etch-and-rinse	3/2/7 2/3/7	1/2/9 0/4/8
SP-3%HP/self-etch SP-3%HP/etch-and-rinse	3/2/7 1/4/7	1/3/8 0/4/8
35%HP gel/self-etch 35%HP gel/etch-and-rinse	2/2/8 1/1/10	2/1/9 0/2/10

Abbreviations: HP, hydrogen peroxide; SP, sodium perborate.

## Data Availability

The datasets generated and/or analyzed during the current study are available from the corresponding author upon reasonable request.
